# Linking the Flint Food Store Survey: Is Objective or Perceived Access to Healthy Foods Associated with Glycemic Control in Patients with Type 2 Diabetes?

**DOI:** 10.3390/ijerph181910080

**Published:** 2021-09-25

**Authors:** Richard Casey Sadler, Amanda Y. Kong, Zachary Buchalski, Erika Renee Chanderraj, Laura A. Carravallah

**Affiliations:** 1 Division of Public Health, Michigan State University, 200 E 1st St., Flint, MI 48502, USA; buchal13@msu.edu (Z.B.); carraval@msu.edu (L.A.C.); 2 Department of Health Behavior, Gillings School of Global Public Health, University of North Carolina, 135 Dauer Drive, Chapel Hill, NC 27599, USA; akong2@live.unc.edu; 3 Department of Nutritional Sciences, University of Michigan School of Public Health, Ann Arbor, MI 48109, USA; eshaver@umich.edu

**Keywords:** geographic information systems, diabetes mellitus, food insecurity, nutrition assessment, urban health

## Abstract

Type 2 diabetes mellitus (DM-2) remains a significant public health concern, particularly in low-income neighborhoods where healthy foods may be scarcer. Despite the well-known relationship between diet and diabetes, little evidence exists on the connections among the objectively measured community and consumer food environment, perception of food access, and diabetes management or outcomes. This cross-sectional, ecological study represents the first example of combining a GIS-based, objectively measured food store audit considering quality, variety, and price of foods in stores with a clinical survey of patients with DM-2 (*n* = 126). In this way, we offer evidence on the relationship between healthy food access—measured more robustly than proximity to or density of certain store types—and diabetes management knowledge, medication adherence, and glycemic control. Better glycemic control was not correlated with better overall food store score, meaning that people in neighborhoods with better access to healthy foods are not necessarily more likely to manage their diabetes. While perceived healthy food access was not correlated with glycemic control, it was strongly correlated with objective healthy food access at shorter distances from home. These results have great importance both for clinical understanding of the persistence of poor diabetes management outcomes and for the understanding of the influence of the food environment on health behaviors.

## 1. Introduction

Although research exists on community food environments and their presumed association with health outcomes [1]—including type 2 diabetes mellitus (DM-2) [2]—limitations in data quality or spatial analytical approaches have led researchers to question which relationships are merely artifacts of study design.

The relationship between food insecurity itself and DM-2 is well-established, but research has not yet provided rigorous approaches to objectively measuring food environments that can be linked via GIS to individual health outcomes, such as DM-2. Indeed, only a few papers (such as [3]) collect objective measures of the food environment, but such studies have not linked GIS-based measures to health outcomes. In this paper, we explore the relationship between objectively measured food access and DM-2-related outcomes, such as HbA1c levels (blood glucose).

Type 2 diabetes mellitus (DM-2) remains the 7th leading cause of death in the US [4]. It is responsible for a range of morbidities, such as renal disease, amputations, and cancers [5], and increased in prevalence from 9.8% to 12.4% from 1994 to 2012 [6]. The relationship between food insecurity and DM-2 is well established. HbA1c is often higher among African American and food-insecure respondents, which may lead to increased difficulty in following a diabetes-appropriate diet and decreased capacity for successful diabetes self-management [7,8,9,10]. In turn, food security is connected to several risk factors and correlates of DM-2, including inflammation, obesity, and insulin resistance [11]. Even when adjusting for depression, diabetes distress, and medication adherence, food insecurity was still a significant contributing risk factor for uncontrolled diabetes [12].

One measure of food access is the concentration or number of food retailers (e.g., grocery stores) within a certain distance or geographic area where an individual resides. The basic idea behind this assumption is that the nutrition environment around one’s home is predictive of where one would routinely shop and, therefore, what they would eat [13,14]. For example, proximity to grocery stores (as defined by industry codes) has been linked to the greater consumption of healthy foods (as in [15]).

A major limitation of these and other studies is that they measure food access by only considering the number or concentration of retailers that sell food products (‘subjective food access’) without considering the quality, variety, or pricing of food products in stores (e.g., measures of *objective* food access that also take into consideration the characteristics of products sold and their pricing may better capture ‘healthy food access’). A neighborhood could have a high number of food retailers, but the variety of healthy products may be low or highly priced; subjective measures of healthy food access cannot capture these important nuances. Since perceived food access is not always concordant with objective food access [16]—both of which in turn can negatively influence DM-2 outcomes—better understanding access to a healthy food environment represents a critical link in the causal chain connecting diabetes to the built environment. Indeed, only a few papers (such as [3]) collect objective measures of the food environment, but such studies have not linked GIS-based measures to specific health outcomes.

In this paper, we explore the relationship between objectively measured healthy food access and DM-2-related outcomes, such as blood glucose (HbA1c) levels in a cross-sectional, ecological study of diabetic patients. Our study site of Flint, Michigan, is exemplary in terms of understanding inequities in healthy food access. Years of disinvestment have meant the departure of most large grocery stores, and the stores that remain have foods of poorer quality, variety, and price [17]. This is particularly the case in poorer and minority neighborhoods. As the first to make this connection between the objectively measured, GIS-based community food environment and DM-2, we aim to build the evidence base on their association, providing us with better tools in advocacy toward healthier built environments.

## 2. Materials and Methods

Our study leverages two datasets: (1) a GIS-based, objectively measured food store survey of stores in the corresponding geographic region, and (2) a clinical survey of diabetes management and health outcomes of DM-2 patients.

### 2.1. Objective Healthy Food Access Scores

We incorporated the Flint Food Store Survey (FFSS) (developed in [17]) to measure consumer- and community-level food availability, or what we refer to as objective healthy food access. The FFSS was directly derived from the Nutrition Environment Measures Survey in Stores (NEMS-S), a valid and reliable tool for measuring the quality, variety, and price of healthy foods in stores [1], which has been used in previous work [18]. The FFSS—completed in September 2016—included assessments of every food retailer in and within two miles of the city of Flint, Michigan (*n* = 265).

We calculated several measures of objective healthy food access. Based on FFSS results, each store was assigned objective food access scores (derived from the quality, variety, and price of healthy foods), both overall and for several subcategories including fruits, vegetables, dairy, meat, and grains. NEMS-S scores ranged from 0 to 91, with higher scores denoting better quality, variety, and price of healthy foods. Using ArcGIS 10.3 (Environmental Systems Research Institute, Redlands, CA, USA, 2018), we geocoded the exact location of every store and appended its corresponding FFSS scores.

We used several metrics to link our NEMS-S scores to the GIS environment. In part, we built on the recommendation to use kernel density estimation (KDE) to better understand the distribution of healthy foods (as opposed to merely the location of healthy food sources) [19,20]. KDE has been shown to be a useful way of representing complex exposure estimates in space when given point-level data [21], but has not been used extensively in food access studies. As one part of our analyses, we, therefore, used the above overall and sub-category scores in a series of KDEs to create surfaces of objective healthy food accessibility for every location in the City of Flint using ArcGIS and determine whether this measure had value in estimating objective healthy food access. We also computed the number of stores with an overall NEMS-S score or an NEMS-S fruit and vegetable score above certain thresholds (these scores represented natural breaks in the data above, and generally represented major chain retailers) within a certain distance (½ mile, 1 mile, and 2 miles network distance) from the patient address. 

### 2.2. Diabetes Survey

We collected surveys from patients with DM-2 at various low-income clinical settings (*n* = 126) from November 2016 to March 2017. Collecting these datasets concurrently minimized temporal mismatch in the nature of the food environment. Survey measures included: perceived availability of healthy foods, most recent HbA1c level (to assess level of glycemic control), address (to link their neighborhood-level food availability in GIS), sociodemographic covariates associated with glycemic control (age, sex, race, education level, and income bracket) and survey questions capturing self-reported management of glycemic health.

#### 2.2.1. Participants

Study participants were 18 years or older with a diagnosis of DM-2 who visit internal medicine, family medicine, or combined internal medicine-pediatric residency clinics in Flint, Michigan, for either diabetic or non-diabetic reasons. These included clinics associated with Hurley Medical Center, McLaren Flint, Genesys Regional Medical Center, and Hamilton Community Health Network. We obtained ethics approval from institutional review boards connected to each of these institutions prior to study participant enrollment.

To screen only Flint area residents, we limited our initial sample to patients with a ZIP code of 48501 to 48507 or 48532. Excluded subjects were those under 18 years old, pregnant women, those who were unable to speak English fluently, those who did not have an HbA1c reading measured within the past 12 months, and those with non-Flint ZIP codes. We addressed the issue of potential error created by only using ZIP codes when studying Flint residents [21,22] by geocoding all addresses to confirm their location inside or outside of our food store survey zone (which was limited to the city limits of Flint).

#### 2.2.2. Data Collection

The clinical survey was conducted at the primary care offices noted above. Medical students distributed a survey to a convenience sample of patients with DM-2 while they awaited discharge instructions (or at another appropriate time as determined by the physicians). At one clinic (Hurley Diabetes Center), nursing staff agreed to administer surveys on the primary research team’s behalf. The researchers or nursing staff explained the project to the patients and obtained consent to collect health information from their charts, including HbA1c; BMI; insulin use; and the presence of comorbidities, such as hypertension and hyperlipidemia.

Medical students also asked a series of questions about perceived food availability, food insecurity, and diabetes self-management to the patient (Appendix A). The questions on the survey were adapted from three peer-reviewed sources: the USDA food insecurity questionnaire, a food availability survey used by several studies [23,24,25], and the Modified Morisky Medication Adherence Scale for DM-2 [26]. Questions addressing diabetes self-management were incorporated from a survey on www.diabetesinitiative.org. Validated survey questions were altered only to account for and reflect the presence of local food resources. The survey also included information about patient demographics, such as education, sex, income level, and address.

### 2.3. Analysis

Due to the large number of potential outcome measures, analysis was performed in two parts. First, the seventeen estimated measures of food access were compared to our participants’ perceived food availability (using Spearman’s correlation tests and adjusted *p*-values) to attempt to identify measures that most accurately captured the food environment. Pursuant to this, the measure that most accurately matched the objective food environment was used as a predictor of HbA1c for our participants in a linear regression model, along with other sociodemographic information and data related to glycemic control. A key strength of this study is that we sought out an objective measure that was most closely correlated with perceived food access, rather than pre-selecting a measure with little or no relevance to the participants. This measure can, therefore, be considered as more closely aligned not only with an objective reality, but how that reality is experienced by participants overall.

## 3. Results

**Figure 1:**
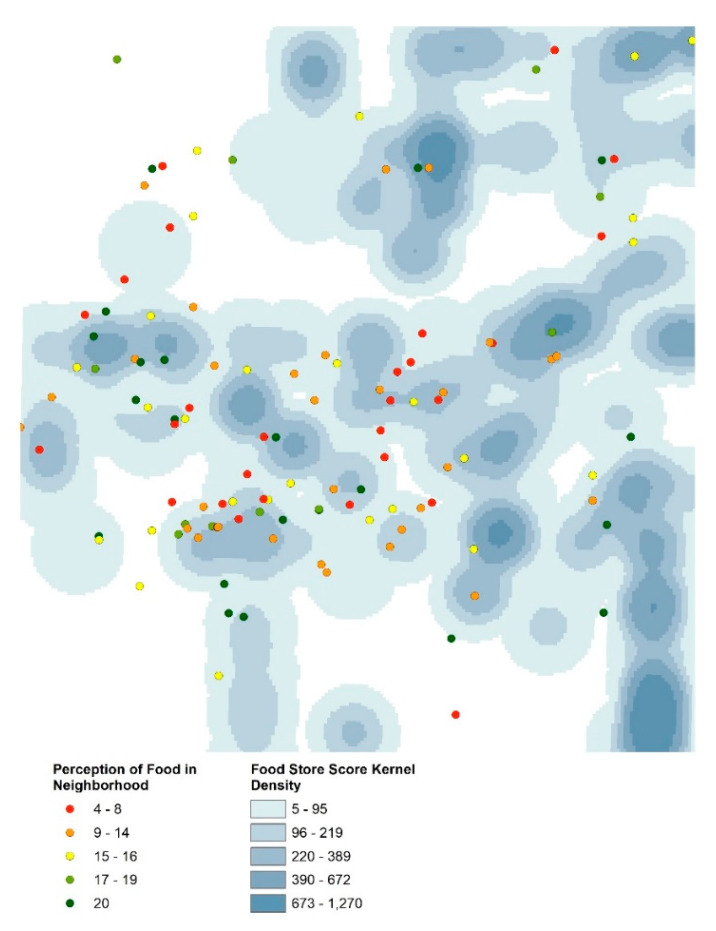
Jittered home locations of respondents (colored by perceived food access) and objective food access (illustrated by kernel density estimation of store scores).

To illustrate how individual characteristics were combined with GIS-based food store scores, Figure 1 highlights anonymized participant location colored by perception of food in their neighborhood overlaid on a KDE surface of objective healthy food access. For each food access metric, scores were extracted from the corresponding geographic layer and appended to the individual’s home location.

Table 1 shows descriptive statistics on the diabetes survey. Self-identified demographics revealed that 60% of the sample were female, 85% were African American, and all lived within the 2-mile buffer zone of the City of Flint. In terms of education, 55% had a high school diploma or less, while only 6.4% had a Bachelor’s degree or higher. In terms of socioeconomic status, 52% lived on less than USD 10,000 a year, 60% used food assistance, and 79% had at least some difficulty living on their household income. Health-wise, 21% stated they had poor health, and 64% used Medicaid, but only 15% claimed a cost barrier to seeing a physician. In terms of self-assessed diabetes knowledge, 60% had taken a diabetes management course, 52% maintained they had good or excellent diabetes knowledge, and 75% reported they did home blood glucose monitoring. 

The results from the comparison between objective healthy food access measures and self-assessed/perceived food access are available in Table 2. Ultimately, the measure that had the highest correlation was the number of NEMS scores > 70 within ½ mile (essentially, a major grocery store nearby). This measure was also one of only two significantly associated measure via a Spearman’s correlation test. To control for multiple comparisons, a Benjamini–Hochberg *p*-value correction was used. As these measures are so similar, we report only the most significant indicators of perceived environment: the number of grocery stores within ½ mile with a NEMS-S score >70 or a modified NEMS-S score > 61.

To explore the overall relationship between HbA1c and food availability in the sample, a regression analysis was run between HbA1c and the two metrics of food availability significantly correlated with perceived food availability. Since these metrics were count variables, they were log-transformed so that the residuals of the linear model would more closely follow a normal distribution. Three nested models were used to ascertain the effect of the food environment on glycemic health (Model 1), the effect of the food environment along with sociodemographic covariates on glycemic health (Model 2), and a model including all of these measures and the measures of self-reported glycemic health management (Model 3). The results are available in Table 3.

Objective healthy food access (as we defined it in our models) is positively associated with HbA1c, though not reaching the level of statistical significance. Among the sociodemographic variables, only age is significantly associated with HbA1c, such that the older members of this population have better glycemic health. We found no significant association with race, but the White population had higher A1C in the model with no indicators of diabetes management, while the coefficient was essentially zero when the management variables were included. This suggests that potential due to race may be a result of an unequal response to lifestyle changes. In addition, the lack of a significant influence of income or education on HbA1C is surprising, though those with more education and a higher income did have slightly lower HbA1C.

None of the individual measures of diabetes management were significantly associated with better glycemic health, though the inclusion of these variables led to a significantly more predictive model (F_4, 89_ = 30.4, *p* < 0.001), suggesting that diabetes management and education are an important part of helping diabetics manage their glycemic health. Among the models added in the third iteration, no associations were significant, although those with the greatest influence on the model were the Modified Morisky Medication Adherence and adherence in the past week (measured as the number of days an individual took their medication and the number of days they took their insulin in a week). Education had essentially no influence, suggesting that it may not be a successful method of improving their HbA1C.

## 4. Discussion

This is the first study to combine GIS-based objective food availability derived from the NEMS-S with HbA1c, providing the first insights into the relationship between the objective food environment and diabetes-related health issues. Key findings include (1) a strong relationship between indicators of improved diabetes management and glycemic health, (2) a correlation between proximity to food stores and better perceived access and (3) a lack of a relationship between perceived access and glycemic control. 

First, the correlation between measures of diabetes management/education and better glycemic control suggests that—more than the neighborhood food environment—simply being educated about best practices for managing diabetes is critical to maintaining healthy HbA1c. Additionally, the inversion in the relationship between proximity to stores and perceived access at greater distances demonstrates a role for continued education about the food environment. Lastly, the lack of a relationship between perception of food availability and glycemic control indicates that there may be a lack of knowledge about quality or availability of food that could be important in shaping health outcomes. 

We used objective measures to define the quality, variety, and price of healthy foods at the neighborhood level, yet our measures were not correlated with the diabetes-related behaviors or outcomes examined. This contrasts with past work that found food access influenced the effect of a diet-based intervention [27]. This alone suggests proximity to grocery stores (at least in the way they have been conceptualized) may not be the best way to measure food environments. Further, the fact that simple count measures of the number of stores above a certain NEMS-S score threshold were the only objective measures to significantly correlate to perceived food access could suggest that—in this sample—individuals’ perceptions of their food environment are strongly shaped by the presence or absence of large, high-quality stores close by. Furthermore, the absence of a significant connection between these food access measures and HbA1c or diabetes management could suggest that the food environment may most suitably be defined at less fine-grained levels (e.g., that access within short distances plays a smaller role in shaping diet than may be expected). Optimistically, this suggests that future work limited by simple measures of counts of grocery stores may be useful in approximating the food environment—at least insofar as they correlate to perceptions.

Our work provides further evidence on the importance of reducing disparities in access to healthy foods: the gaps in Flint’s food environment have been studied extensively, and our highly distressed sample generally lived in neighborhoods with no chain grocery stores or farmers’ markets, where groceries tended to be more expensive than elsewhere in the region and where other samples have yielded below-average quality diets [17,28,29,30]. Within Flint, in particular, the combination of increases in poverty and decreases in healthy food access have created a population highly vulnerable to diet-related disease. While our research fails to find evidence that access to healthy food improves health for diabetics, we were deliberate in our approach to measuring the food environment. As Flint is already an economically depressed, food-sparse area, it may be that proximity to healthy food is not the best way to measure the impact of healthy food on community health. However, just as stakeholders have quickly started using the FFSS to address gaps in the food environment (discussed in [17]), others can also use the methods and findings here to advocate for further improvements in study design and policy advocacy. 

### Limitations and Future Directions

We acknowledge limitations arising from our adapted NEMS-S survey and the spatial analysis technique employed to derive food access scores. First, while the NEMS-S is the recommended method for understanding exposure or access in geographic research (as in [20]), the use of KDE in studies of NEMS-S-based food access remains underdeveloped, does not perfectly represent the food environment in which a person shops, and was not significantly correlated with perceived food access in our sample. It may be that KDE is not a suitable way to represent the food environment, but further studies could explore this further to gain a better understanding. Any representation of food access is a simplification of the real world; ultimately, most people do not shop solely within their home neighborhoods. Additionally, while home environments serve as a fairly suitable proxy for exposure [13], activity spaces extend to other locations, such as workplaces, schools, places of worship, and social networks [31].

Further, we used a convenience sample from a predominately low-income population (64% were Medicaid recipients, and 57% made less than USD 10,000), which complicates the generalizability of our findings. Reflecting this and the fact that our sample was collected from diabetes clinics, the mean HbA1c found in our study (7.87) was higher than the mean (7.20) and 75th percentile (7.80) in the NHANES 2004. Lastly, our study had low power (*n* = 126 participants meeting inclusion criteria), which may have prevented the detection of statistically significant associations.

Despite these limitations, however, as the first study to link an objective measurement of the food environment in a GIS with a clinical survey of health outcomes, we provide powerful new evidence for studies linking the food environment and health. GIS has been used to help map disparities in rates of diabetes and target areas for response [32,33], but this additional level—of linking diabetes management with objective healthy food access—opens the door for many new areas of intervention. Furthermore, as the world has experienced since March 2020, comorbidities such as diabetes strongly influence the severity of the body’s reaction to COVID-19 [34,35,36]. This work, therefore, has added relevance as practitioners and researchers seek to uncover the landscapes where people are more vulnerable to this and other related diseases. Each of these points provide further reason for policy advocacy toward land use design that encourages mixed land uses and the amelioration of so-called ‘food deserts’. Reducing such disparities should, in theory, diminish health inequities arising from poor access to healthy foods.

## 5. Conclusions

Next steps include future studies with a larger and more varied sample of subjects to further explore whether these GIS-based measures have utility in linking to dietary behaviors or outcomes. One such opportunity is derived from accompanying work in Flint around the response to the Flint Water Crisis: the Hurley Pediatric Clinic has been delivering fruit and vegetable prescriptions to pediatric patients in recent years, and preliminary work suggests a strong link between those programs and diet [30,37,38]. Our team is currently exploring links between this program and the food access measures used here. Considerable future opportunities also exist with respect to this dataset and other health outcomes, including using GIS-based data to explore the relationship between food availability and other chronic diseases, such as hypertension and obesity. Lastly, completing an equivalent food store survey in other communities would provide the opportunity to determine whether these associations hold in other places, and give other local stakeholders in-depth information on their food environment that would not be possible otherwise. Such knowledge can help target spatially explicit food retail interventions and health messaging programs, as the researchers have a better idea of where specific disparities in access exist. More broadly, the opportunity to replicate this study in other communities and with other health outcomes offers a rich future line of work from which researchers can gain a much deeper understanding into the relationship between the built environment and health.

## Figures and Tables

**Table 1:** Summary statistics.

	Overall
*N*	160
Age (mean (SD))	55.46 (11.76)
Sex = Female (%)	101 (63.1)
Race (%)	
African American	123 (77.8)
Caucasian	35 (22.2
Education (%)	
Less Than High School	32 (20.0)
High School	57 (35.6)
More Than High School	71 (44.4)
Income Bracket (%)	
Under USD 10k	85 (57.4)
USD 10k to USD 25k	46 (31.1)
Over USD 25k	17 (11.5)
Government Assistance for Food = No (%)	62 (38.8)
Difficulty Living on Household Income (%)	
Not Difficult	34 (21.2)
Somewhat Difficult	43 (26.9)
Difficult	42 (26.2)
Very Difficult	27 (16.9)
Extremely Difficult	14 (8.8)
Time since diagnosis (years) (mean (SD))	11.52 (10.28)
Exercise (days/week) (mean (SD))	2.02 (2.49)
Modified Morisky Medication Adherence (4 questions; 1—yes; 0—no; possible—0–4) (mean (SD))	1.01 (1.06)
Self-care (7 questions; 1—yes; 0—no; possible—0–7) (mean (SD))	4.41 (1.58)
Perception of food in the neighborhood (4 questions; possible –1–5; 5–20) (mean (SD))	13.78 (5.17)
Food insecurity total (5 questions, often/sometimes/yes—1; never/no—5; possible—0–5 (mean (SD))	1.83 (2.03)
General Health (%)	
Excellent	4 (2.5)
Good	34 (21.2)
Fair	88 (55.0)
Poor	34 (21.2)
Medicaid = No (%)	58 (36.2)
Food Insecure = No (%)	90 (56.2)
Transportation Issues = No (%)	23 (71.9)

**Table 2:** Association between objective and perceived food access.

Food Access Variable	Spearman’s Rho	*p*-Value (Corrected)
KDE of NEMS store scores within 1 mile	0.057	0.524 (0.920)
KDE of modified store scores (using alternate F&V measure #2 within 1 mile)	0.051	0.567 (0.920)
KDE of modified store scores (using alternate F&V measure #3 within 1 mile)	0.050	0.575 (0.920)
Number of stores with a standard NEMS score >70 within ½ mile	0.265	0.003 (0.034)
Number of stores with a modified NEMS score >61 within ½ mile	0.253	0.004 (0.034)
Average store score of stores within 1/2 mile	−0.007	0.942 (0.985)
Average alternate measure #2 of stores within 1/2 mile	0.003	0.972 (0.985)
Average alternate measure #3 of stores within 1/2 mile	−0.015	0.867 (0.920)
Number of stores with a standard NEMS score >70 within 1 mile	0.075	0.406 (0.920)
Number of stores with a modified NEMS score >61 within 1 mile	0.077	0.393 (0.920)
Average store score of stores within 1 mile	−0.010	0.906 (0.985)
Average alternate measure #2 of stores within 1 mile	0.002	0.985 (0.985)
Average alternate measure #3 of stores within 1 mile	−0.017	0.848 (0.920)
Average store score of stores within 2 miles	−0.081	0.367 (0.920)
Average alternate measure #2 of stores within 2 miles	−0.094	0.295 (0.920)
Average alternate measure #3 of stores within 2 miles	−0.081	0.370 (0.920)

Underline denotes significance.

**Table 3:** Regression analysis.

	Model 1	Model 2	Model 3
(Intercept)	8.302 ***	11.313 ***	9.638 ***
	[7.860,8.743]	[8.679,13.948]	[6.238,13.037]
Number of stores within a half-mile with NEMS Score > 70	0.400	0.507	0.620
	[−1.285,2.085]	[−1.207,2.221]	[−1.175,2.414]
Age		−0.049 **	−0.044 *
		[−0.084,−0.013]	[−0.083,−0.005]
Sex (Reference: Male)		−0.201	−0.351
		[−1.086,0.683]	[−1.290,0.587]
Race (Reference: African American)		0.443	−0.046
		[−0.781,1.668]	[−1.375,1.284]
**Education (Reference: Less than High School)**			
Education: High School		−0.387	−0.257
		[−1.583,0.808]	[−1.540,1.025]
Education: More than High School		−0.433	−0.203
		[−1.624,0.757]	[−1.506,1.100]
**Income Bracket (Reference: Under USD 10k)**			
Income Bracket: 10k to 25k		−0.036	−0.015
		[−1.059,0.988]	[−1.101,1.070]
Income Bracket: Over 25k		−0.895	−1.072
		[−2.313,0.524]	[−2.583,0.440]
Time since diagnosis (years)		0.032	0.017
		[−0.009,0.074]	[−0.028,0.063]
Self-Reported Diabetes Knowledge			0.068
			[−0.517,0.653]
Adherence Last Week (0–14 points)			0.105
			[−0.010,0.220]
Modified Morisky Medication Adherence			0.338
			[−0.104,0.780]
Self-Care (0–7 points)			0.109
			[−0.216,0.433]
*N*	119	107	101
R-squared	0.002	0.118	0.171

*** *p* < 0.001; ** *p* < 0.01; * *p* < 0.05.

## Data Availability

Anonymized, aggregated data can be made available upon request.

## Appendix A. Diabetes Management Research Survey

**Patient Demographics**

Age

Sex

Race

Address

Education level (circle one) (Elementary, High School, Some College, Associate’s, Bachelor’s, Graduate)

Income (Less than USD 10,000, USD 10,001–USD 24,999, USD 25,000 or more, Decline to answer)

Do you receive government assistance for food (e.g., SNAP EBT WIC)? (Yes, No)

How difficult is it for you to live on your total household income right now?

(Not at all difficult, Somewhat difficult, Difficult, Very difficult, Extremely difficult)

**General Health & Healthcare Access**

Would you say that in general your health is? (Excellent, Good, Fair, Poor)

Do you have health insurance coverage through (Mark all that apply) (Employer, Medicaid, Medicare, Self Pay, Uninsured, Genesee County Health Plan, Other)

Was there a time in the past 12 months when you needed to see a doctor but could not because of cost? (Yes, No)

**Diabetes Management**

How long have you been diagnosed with diabetes? (Yes, No)

Have you ever taken a course or class in how to manage your diabetes yourself?

I feel my knowledge on diabetes is (Excellent, Good, Fair, Poor)

Do you take your blood sugar readings at home? (Yes, No)

How many times in an average week do you exercise for at least 30 min? (0–7)

Do you take any diabetes medication or insulin? (Medication, Insulin, None)

In the last week, how many days did you take your recommended diabetes medications? (0–7)

In the last week, how many days did you take your recommended insulin injections? (0–7)

Do you ever forget to take your Diabetes medicine? (Yes, No)

Do you ever have problems remembering to take your Diabetes medication? (Yes, No)

When you feel better, do you sometimes stop taking your Diabetes medication? (Yes, No)

Sometimes if you feel worse when you take your Diabetes medicine, do you stop taking it? (Yes, No)

I have had my **clinical foot examination** within the past year. (Yes, No)

I have had an **eye exam** within the past year. (Yes, No)

I have had a **dental visit** within the past year. (Yes, No)

I have been tested for microalbuminuria **(urine) test** within the past year. (Yes, No)

I have received my **flu shot** within the past year. (Yes, No)

I have received a **pneumonia shot**. (Yes, No)

I have received my **HbA1C** (average blood sugar) test regularly. (Yes, No)

**Food Availability**

How much difficulty do you have getting to a grocery store or supermarket that has a good variety of fresh fruits and vegetables? (No difficulty at all, Not much difficulty, Some difficulty, A lot of difficulty)

A large selection of fresh fruits and vegetables are available in my neighborhood (Strongly Disagree, Disagree, Neutral, Agree, Strongly Agree)

The fresh fruits and vegetables in my neighborhood are of high quality (Strongly Disagree, Disagree, Neutral, Agree, Strongly Agree)

A large selection of low fat products is available in my neighborhood (Strongly Disagree, Disagree, Neutral, Agree, Strongly Agree)

There is access to adequate food shopping in your neighborhood (Strongly Disagree, Disagree, Neutral, Agree, Strongly Agree)

Are the following present in your neighborhood? (neighborhood = 20 min walk or 1 mile from your house)

A supercenter, such as Wal-Mart, Meijer, or Target (Yes, No)

A supermarket, such as Aldi, Kroger, Mr. B’s, or Landmark (Yes, No)

A smaller grocery store (Yes, No)

A convenience store with or without a gas station attached (Yes, No)

A specialty store, such as ethnic, meat market, seafood market, green grocer, or bakery (Yes, No)

A freestanding drug store or pharmacy, such as CVS, Walgreens, or Rite-Aid (Yes, No)

A dollar variety, dollar general, dollar store, or dollar tree (Yes, No)

A franchised fast food restaurant, including McDonald’s or Subway (Yes, No)

A sit down restaurant or buffet restaurant (Yes, No)

**Food Insecurity**

In the last 12 months, how often were these statements true?

The food that I bought just did not last, and I did not have money to get more.” (Often true, Sometimes true, Never true, Unsure or Refused)

“I could not afford to eat balanced meals.” (Often true, Sometimes true, Never true, Unsure or Refused)

Did you or other adults in your household ever cut the size of your meals or skip meals because there was not enough money for food? (Yes, No, Unsure)

[IF YES ABOVE, ASK] How often did this happen? (Almost every month, Some months but not every month, Only 1 or 2 months, Unsure)

Did you ever eat less than you felt you should because there was not enough money for food? (Yes, No, Unsure)

Were you ever hungry but did not eat because there was not enough money for food? (Yes, No, Unsure)
